# Expression of Concern: Differential microRNA profiling of the Marshallese population in Arkansas reveals a higher association with chronic diseases

**DOI:** 10.1371/journal.pone.0352624

**Published:** 2026-06-30

**Authors:** The PLOS One Editors

After this article [1] was published, concerns were noted that the articles cited as References 59, 76, 78, and 81 were retracted before [1] was published.

In light of these concerns, the *PLOS One* Editors issue this Expression of Concern. Readers are advised to interpret the article [1] with caution.
