# Supplementary figures and images for: Differential microRNA profiling of the Marshallese population in Arkansas reveals a higher association with chronic diseases
Source: PLoS One. 2025 Aug 11;20(8):e0329321. doi: 10.1371/journal.pone.0329321 (PMC12338811; doi:10.1371/journal.pone.0329321)

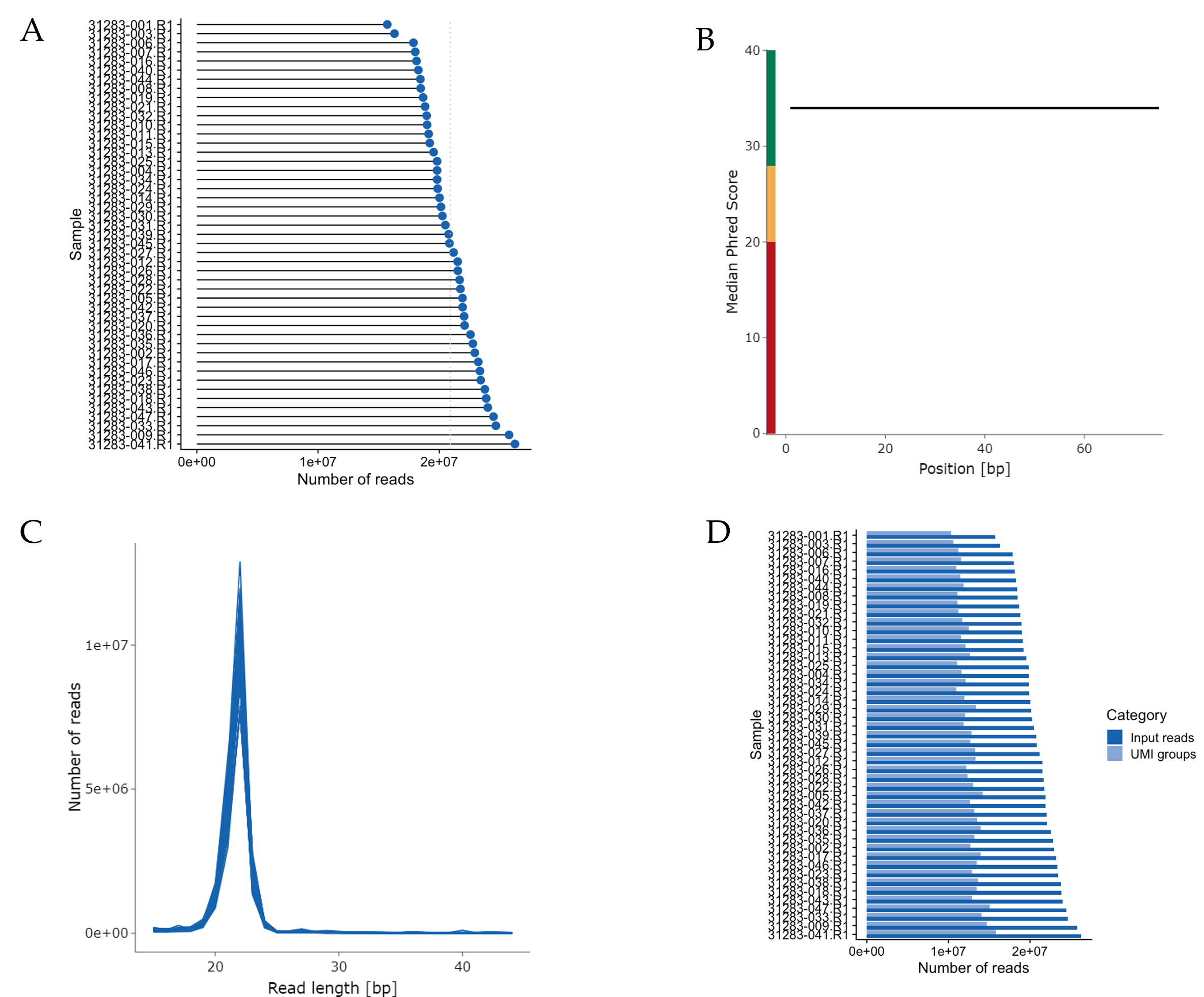

Supplement: S1 Fig — (A) Number of reads per sample. The dotted vertical line indicates the mean number of reads. (B) For each sample, the median Phred quality score over all reads is shown at each read position. The colored bar classifies the Phred values into 3 quality categories: poor (red, 0 to < 20), medium (yellow, 20 to < 28) and good (green, 28 and higher). (C) Distribution of trimmed read length showed a distinct peak at approximately 22 nt, aligning with the expected miRNA length. (D) Read deduplication for each sample with the number of read pairs is plotted (dark blue) in relation to the number of collapsed UMI reads (light blue). (TIF) [file pone.0329321.s001.tif]

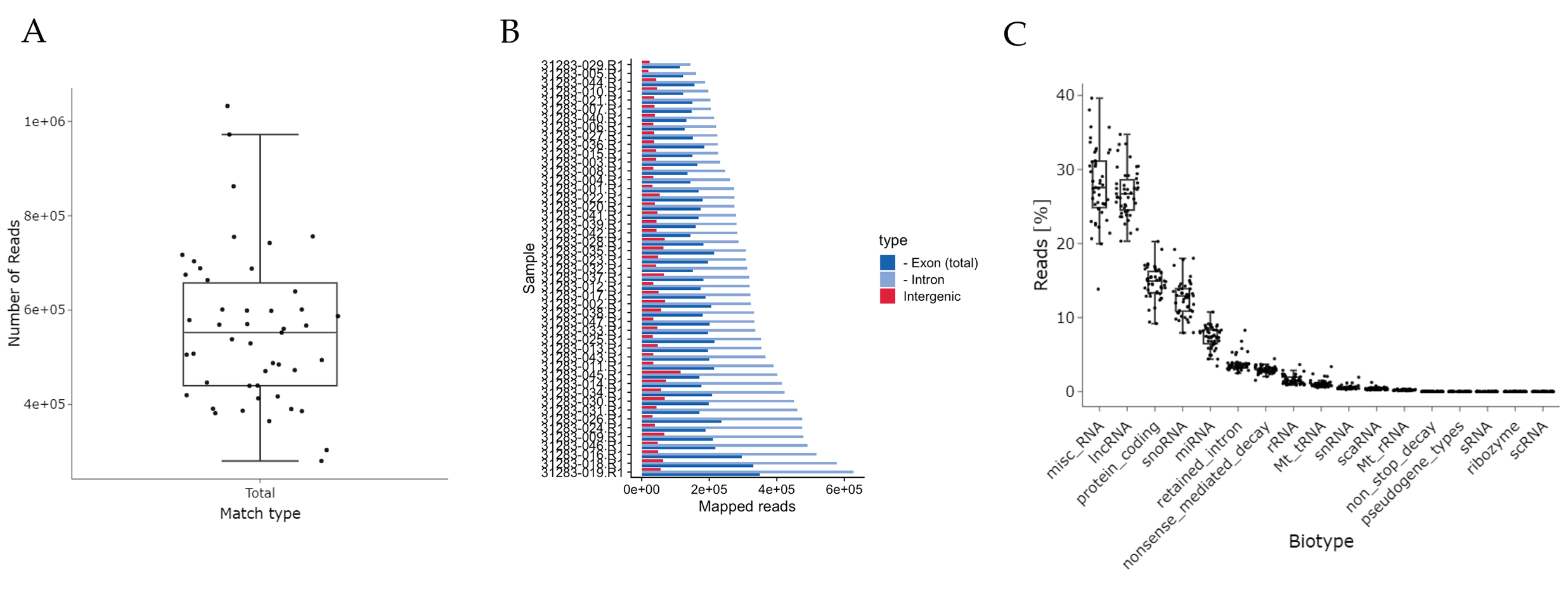

Supplement: S2 Fig — (A) Reads that failed to map to miRBase and other databases of small RNA were mapped to the ENSEMBL annotation. (B) Number of reads mapped to the reference genome split by gene element. (C) Proportion of reads per sample mapped to gene biotypes. (TIF) [file pone.0329321.s002.tif]

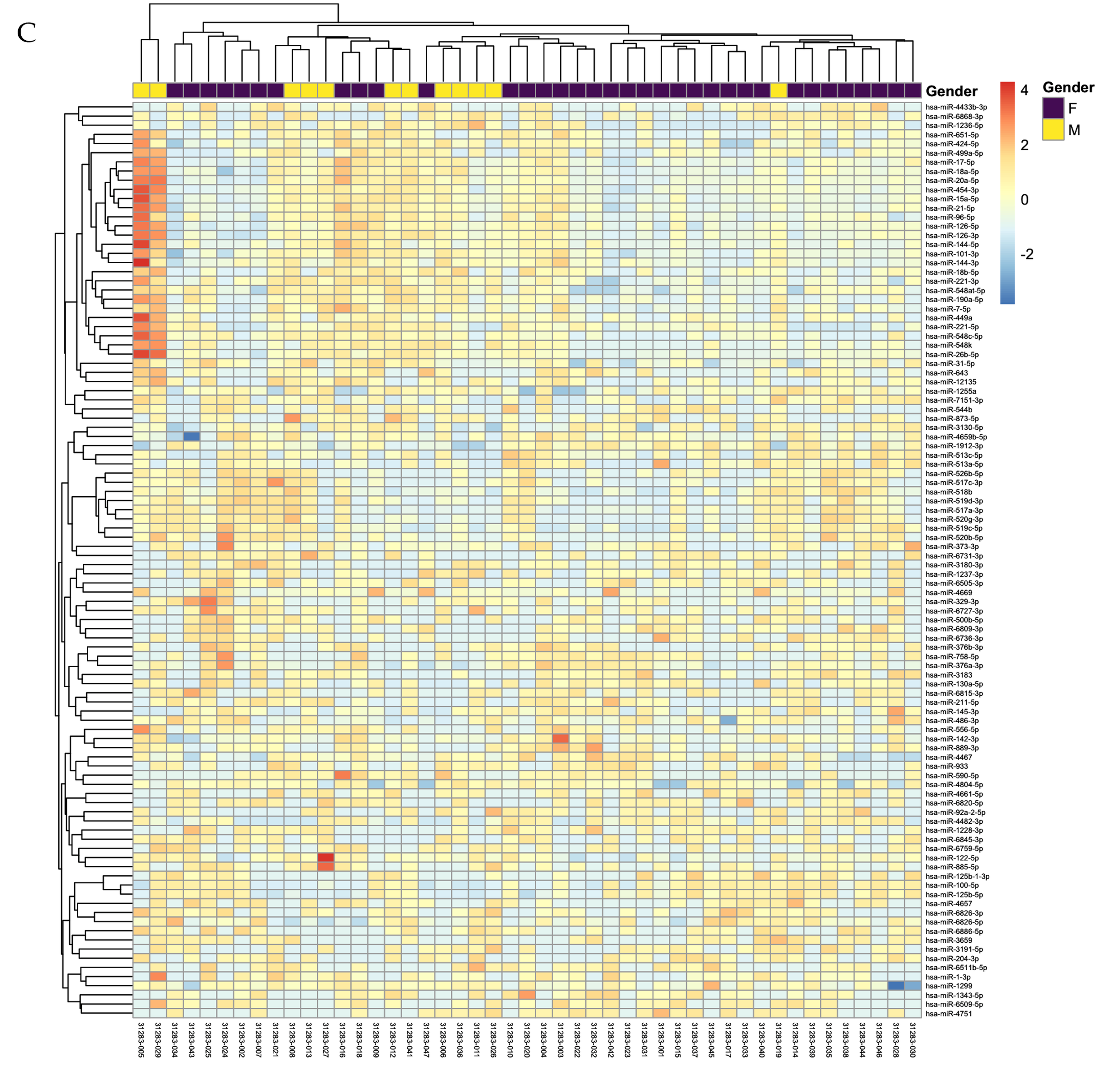

Supplement: S3 Fig — A variance-stabilized transformation was performed on the raw count matrix. Each row represents 1 gene, and each column represents 1 sample. The color represents the difference of the count value to the row mean. (TIF) [file pone.0329321.s003.tif]
