# Supplementary material for: Differential microRNA profiling of the Marshallese population in Arkansas reveals a higher association with chronic diseases
Source: PLoS One. 2025 Aug 11;20(8):e0329321. doi: 10.1371/journal.pone.0329321 (PMC12338811; doi:10.1371/journal.pone.0329321)
Supplement: S1 Table — (DOCX) [file pone.0329321.s004.docx]

**S1 Table.** Most significantly differentially expressed miRNAs in male versus female subjects.

|  | **Name** | **max_group_mean** | **log2FC** | **FC** | ***P*-value** | **FDR** | **Bonferroni** |
| --- | --- | --- | --- | --- | --- | --- | --- |
| **1** | hsa-miR-98-5p | 6,516.50 | 2.21 | 4.62 | 3.70E-15 | 6.00E-12 | 6.00E-12 |
| **2** | hsa-miR-96-5p | 18.58 | 1.34 | 2.54 | 0.00034 | 0.0089 | 0.56 |
| **3** | hsa-miR-95-3p | 11.33 | 1.63 | 3.1 | 9.00E-07 | 0.000049 | 0.0015 |
| **4** | hsa-miR-93-5p | 137,713.17 | 1.22 | 2.33 | 3.20E-07 | 0.000022 | 0.00052 |
| **5** | hsa-miR-7-5p | 5,820.58 | 0.99 | 1.99 | 0.00023 | 0.0063 | 0.37 |
| **6** | hsa-miR-6857-3p | 184.4 | -0.86 | -1.82 | 1.30E-08 | 1.4E-06 | 0.000022 |
| **7** | hsa-miR-6767-5p | 37.49 | -1.85 | -3.6 | 0.00029 | 0.0078 | 0.48 |
| **8** | hsa-miR-641 | 768.09 | -1.84 | -3.58 | 0.000046 | 0.0015 | 0.074 |
| **9** | hsa-miR-590-3p | 3 | 3.63 | 12.34 | 1.4E-06 | 0.000071 | 0.0023 |
| **10** | hsa-miR-584-3p | 33.83 | -0.83 | -1.77 | 0.00018 | 0.0051 | 0.29 |
| **11** | hsa-miR-548l | 5.5 | 2.3 | 4.91 | 3.60E-07 | 0.000022 | 0.00058 |
| **12** | hsa-miR-548k | 4.5 | 2.2 | 4.6 | 9.7E-06 | 0.00038 | 0.016 |
| **13** | hsa-miR-548j-5p | 32.58 | 1.47 | 2.76 | 1.1E-06 | 0.000055 | 0.0017 |
| **14** | hsa-miR-548c-5p | 6.5 | 2.5 | 5.66 | 3.50E-07 | 0.000022 | 0.00057 |
| **15** | hsa-miR-548ax | 2.92 | 2.06 | 4.16 | 0.000096 | 0.0031 | 0.16 |
| **16** | hsa-miR-548au-5p | 1.5 | 2.01 | 4.03 | 0.00014 | 0.004 | 0.22 |
| **17** | hsa-miR-548at-5p | 9.92 | 1.45 | 2.73 | 0.000038 | 0.0013 | 0.061 |
| **18** | hsa-miR-4999-5p | 4 | 3.21 | 9.23 | 1.90E-07 | 0.000015 | 0.00031 |
| **19** | hsa-miR-454-3p | 254.25 | 2.03 | 4.08 | 2.50E-11 | 5.00E-09 | 4.00E-08 |
| **20** | hsa-miR-449a | 6.5 | 2.28 | 4.87 | 6.8E-06 | 0.00029 | 0.011 |
| **21** | hsa-miR-374a-5p | 10 | 2.18 | 4.52 | 1.6E-06 | 0.000077 | 0.0026 |
| **22** | hsa-miR-3682-3p | 13.83 | 1.03 | 2.05 | 0.000068 | 0.0022 | 0.11 |
| **23** | hsa-miR-3613-5p | 3.08 | 2.69 | 6.44 | 8.9E-06 | 0.00036 | 0.014 |
| **24** | hsa-miR-34a-5p | 57.42 | 1.21 | 2.32 | 2.60E-07 | 0.000019 | 0.00042 |
| **25** | hsa-miR-331-3p | 153.94 | -0.62 | -1.53 | 0.0002 | 0.0055 | 0.32 |
| **26** | hsa-miR-323a-3p | 38.66 | -1.13 | -2.18 | 0.00031 | 0.008 | 0.5 |
| **27** | hsa-miR-32-3p | 8.67 | 3.38 | 10.43 | 3.30E-09 | 4.40E-07 | 0.0000053 |
| **28** | hsa-miR-26b-5p | 13,147.67 | 2.03 | 4.09 | 1.10E-12 | 2.90E-10 | 1.70E-09 |
| **29** | hsa-miR-223-5p | 64.5 | 1.82 | 3.52 | 1.60E-08 | 1.6E-06 | 0.000026 |
| **30** | hsa-miR-221-3p | 519.83 | 0.99 | 1.99 | 0.000014 | 0.00053 | 0.023 |
| **31** | hsa-miR-21-5p | 370.08 | 1.78 | 3.45 | 8.20E-07 | 0.000046 | 0.0013 |
| **32** | hsa-miR-20b-5p | 3,685.42 | 1.32 | 2.49 | 2.5E-06 | 0.00012 | 0.0041 |
| **33** | hsa-miR-20a-5p | 1,565.42 | 2.16 | 4.48 | 1.80E-08 | 1.7E-06 | 0.00003 |
| **34** | hsa-miR-199a-5p | 33.23 | -1.04 | -2.06 | 0.0001 | 0.0033 | 0.17 |
| **35** | hsa-miR-199a-3p | 17 | 2.65 | 6.29 | 6.40E-09 | 8.00E-07 | 0.00001 |
| **36** | hsa-miR-196b-5p | 34.92 | 2.1 | 4.29 | 1.30E-08 | 1.4E-06 | 0.00002 |
| **37** | hsa-miR-195-5p | 26.75 | 1.17 | 2.25 | 0.000038 | 0.0013 | 0.062 |
| **38** | hsa-miR-1912-3p | 9.06 | -2.87 | -7.3 | 1.80E-09 | 2.90E-07 | 0.0000029 |
| **39** | hsa-miR-190b-5p | 4.08 | 1.5 | 2.84 | 0.00024 | 0.0065 | 0.39 |
| **40** | hsa-miR-190a-5p | 6.42 | 2.37 | 5.17 | 1.30E-07 | 0.000011 | 0.00021 |
| **41** | hsa-miR-18a-5p | 92.17 | 1.72 | 3.29 | 4.00E-07 | 0.000024 | 0.00065 |
| **42** | hsa-miR-17-5p | 433.83 | 1.17 | 2.25 | 0.000094 | 0.003 | 0.15 |
| **43** | hsa-miR-16-5p | 393,818.67 | 1.19 | 2.28 | 3.10E-07 | 0.000022 | 0.00051 |
| **44** | hsa-miR-15a-5p | 3,113.67 | 2.6 | 6.06 | 1.60E-11 | 3.70E-09 | 2.60E-08 |
| **45** | hsa-miR-144-5p | 8.17 | 2.79 | 6.94 | 0.000008 | 0.00033 | 0.013 |
| **46** | hsa-miR-144-3p | 328.25 | 3.82 | 14.13 | 9.30E-13 | 2.90E-10 | 1.50E-09 |
| **47** | hsa-miR-140-3p | 12,468.29 | -0.56 | -1.47 | 0.00002 | 0.00074 | 0.033 |
| **48** | hsa-miR-139-5p | 1,245.69 | -0.82 | -1.76 | 0.00018 | 0.0051 | 0.29 |
| **49** | hsa-miR-1294 | 933.92 | 0.63 | 1.55 | 0.00011 | 0.0034 | 0.19 |
| **50** | hsa-miR-126-5p | 467.25 | 2.02 | 4.05 | 0.000011 | 0.00044 | 0.018 |
| **51** | hsa-miR-126-3p | 17,538.92 | 2.25 | 4.77 | 3.10E-09 | 4.40E-07 | 0.0000049 |
| **52** | hsa-miR-122-5p | 2,570.83 | 3.22 | 9.29 | 3.40E-13 | 1.80E-10 | 5.50E-10 |
| **53** | hsa-miR-106b-5p | 273.5 | 1.28 | 2.43 | 7.30E-07 | 0.000042 | 0.0012 |
| **54** | hsa-miR-101-3p | 61.42 | 1.71 | 3.27 | 0.00011 | 0.0034 | 0.18 |
| **55** | hsa-let-7i-5p | 307,324.75 | 1.47 | 2.78 | 1.90E-10 | 3.50E-08 | 3.10E-07 |
| **56** | hsa-let-7g-5p | 237,009.08 | 1.82 | 3.53 | 1.30E-13 | 1.10E-10 | 2.10E-10 |
| **57** | hsa-let-7f-5p | 160,157.83 | 1.66 | 3.17 | 4.30E-13 | 1.80E-10 | 7.00E-10 |
| **58** | hsa-let-7e-5p | 439.83 | 0.85 | 1.8 | 5.1E-06 | 0.00022 | 0.0082 |
| **59** | hsa-let-7d-5p | 130,858.33 | 0.8 | 1.74 | 2.60E-07 | 0.000019 | 0.00041 |
| **60** | hsa-let-7c-5p | 1,898.25 | 0.88 | 1.83 | 1.5E-06 | 0.000071 | 0.0024 |
| **61** | hsa-let-7b-5p | 408,869.75 | 0.89 | 1.86 | 3.5E-06 | 0.00016 | 0.0057 |
| **62** | hsa-let-7b-3p | 118.37 | -0.8 | -1.74 | 0.000037 | 0.0013 | 0.06 |
| **63** | hsa-let-7a-5p | 643,482.33 | 0.97 | 1.96 | 2.00E-08 | 1.8E-06 | 0.000033 |

^a^ Log2 fold change and the significant changes are defined as FDR < 0.01.

^b^ FC, fold change; log2FC, log2 fold change; FDR, false discovery rate; Bonferroni, Bonferroni-corrected *P*-value.
